# Supplementary material for: Phosphorylation of tau at a single residue inhibits binding to the E3 ubiquitin ligase, CHIP
Source: Nat Commun. 2024 Sep 12;15:7972. doi: 10.1038/s41467-024-52075-1 (PMC11393453; doi:10.1038/s41467-024-52075-1)
Supplement: Supplementary file 1 — Supplemental Information [file 41467_2024_52075_MOESM1_ESM.pdf]

Supplementary Information

Phosphorylation of Tau at a Single Residue Inhibits Binding to the E3 Ubiquitin Ligase, CHIP

Cory Nadel[1,4], Saugat Pokhrel[1,4], Kristin Wucherer[1], Abby Oehler[3,4], Aye Thwin[2,4], Koli Basu[1], Matthew Callahan[1,4], Daniel R. Southworth[2,4], Daniel A. Mordes[3,4], Charles S. Craik[1], and Jason E. Gestwicki[1,4,*]

Departments of [1]Pharmaceutical Chemistry, [2]Biochemistry & Biophysics and [3]Pathology and the [4]Institute for Neurodegenerative Diseases, University of California San Francisco, San Francisco, CA 94158

Supplemental Methods: Table S1: Details of the crystallography experiments.

Supplemental Fig. S1 Expression of tauC3 in insect cells yields protein that is phosphorylated at multiple sites, including pathogenic residues.
Supplemental Fig. S2. Phosphorylation of Hsp70’s C-terminus limits binding to CHIP and alignment with tauC3’s C-terminus suggests a conserved mechanism.
Supplemental Fig. S3 Pseudo-phosphorylation at serine 416 is sufficient to inhibit CHIP binding.
Supplemental Fig. S4 Mutation of a conserved residue in CHIP (CHIPD134A) partially restores activity on tauC3 pS416.
Supplemental Fig. S5 Additional examples of pS416 accumulation in AD and co-localization with tauC3.
Supplemental Fig. S6. Raw, uncropped western blots and image quantifications for select examples.

SUPPLEMENTAL METHODS; TABLE S1: Details of the crystallography experiments

	CHIP-TPR 10mer tau (r26)
Wavelength	
Resolution range	61.33 - 1.848 (1.914 - 1.848)
Space group	C 1 2 1
Unit cell	82.34 46.0351 70.7818 90 119.95 90
Total reflections	112271 (11324)
Unique reflections	19695 (1931)
Multiplicity	5.7 (5.9)
Completeness %)	98.52 (96.13)
Mean I/sigma (I)	4.24 (1.12)
Wilson B-factor	21.65
R-merge	0.2059 (1.281)
R-meas	0.2266 (1.406)
R-pim	0.09307 (0.5691)
CC1/2	0.986 (0.571)
CC*	0.997 (0.853)
Reflections used in refinement	19580 (1913)
Reflections used for R-free	1006 (96)
R-work	0.2040 (0.3152)
R-free	0.2436 (0.3155)
CC (work)	0.944 (0.787)
CC (free)	0.883 (0.716)
Number of non-hydrogen atoms	2356
Macromolecules	2171
Solvent	185
Protein residues	274
RMS (bonds)	0.008
RMS (angles)	0.85
Ramachandran favored (%)	98.12
Ramachandran allowed (%)	1.88

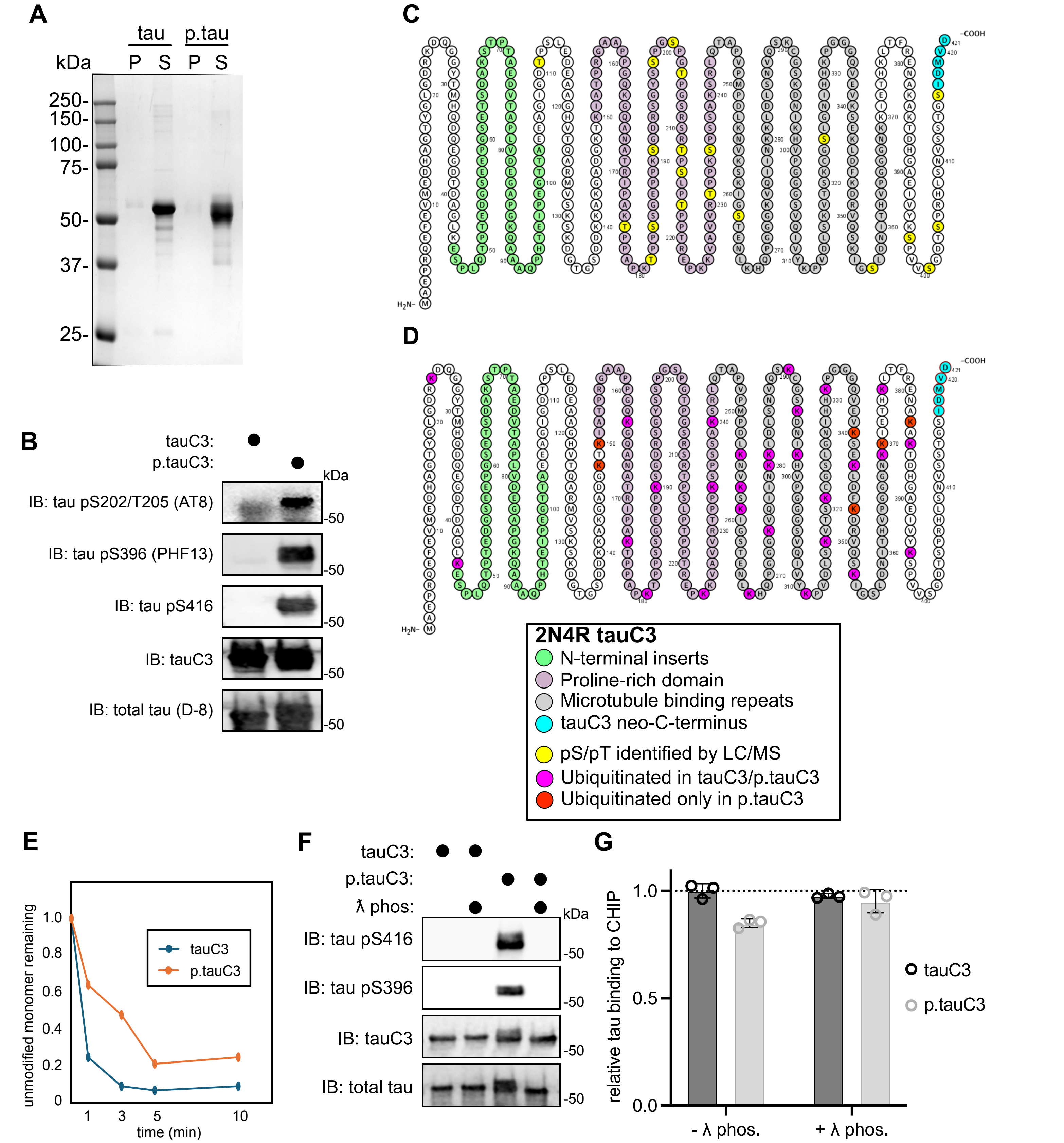

Supplemental Fig S1. Expression of tauC3 in insect cells yields protein that is phosphorylated at multiple sites, including pathogenic residues. **(A)** Representative coomasie-stained gel, showing the purity of tau proteins. **(B)** Western blot confirmation of phospho-epitopes on p.tauC3. The tauC3 and total tau antibodies were used as loading controls. **(C,D)** Cartoons depicting identified sites of (C) phosphorylation on p.tauC3 and (D) ubiquitination on tauC3 or p.tauC3 following in vitro ubiquitination by CHIP. Shared sites for tauC3 and p.tauC3 are depicted in purple, while sites unique to p.tauC3 are shown in red. **(E)** Quantification of ubiquitination rates from Fig 1E. **(F)** Western blot confirming dephosphorylation of p.tauC3 by λ phosphatase. **(G)** TauC3 or p.tauC3 binding to immobilized CHIP following dephosphorylation by λ phosphatase measured by ELISA. Assay was performed in technical riplicate and normalized to tauC3 absorbance at 450 nM without λ phosphatase treatment.

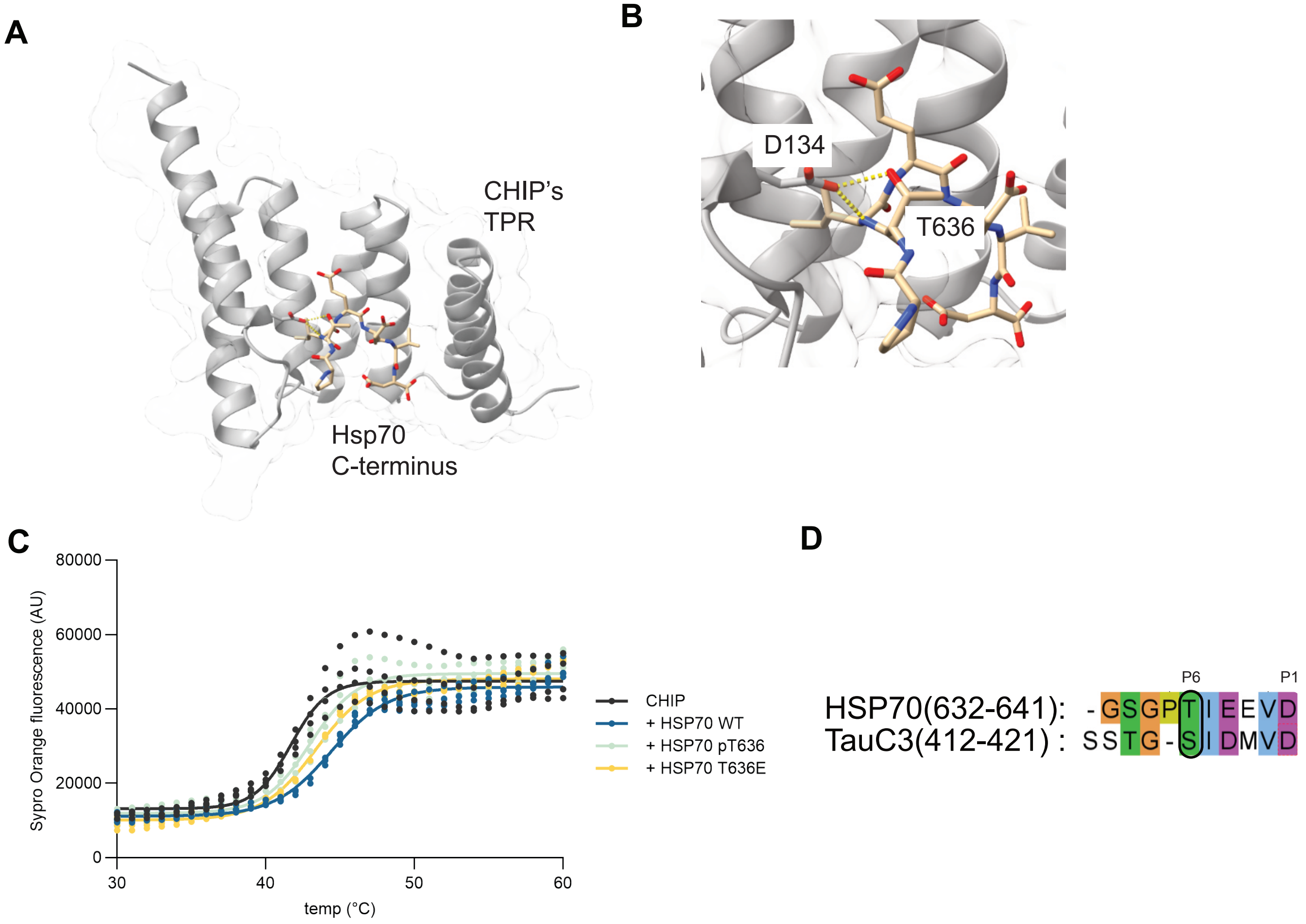

Supplemental Fig. S2. Phosphorylation at a Thr residue in Hsp70's C-termini limits CHIP binding. (A) Binding pose of Hsp70's C-terminal peptide in CHIP's TPR domain (PDB 3Q49). **(B)** Closeup of residue D134 in CHIP and its proximity to Thr636 in Hsp70's C-terminus (P6 position). If this position is phosphorylated, one would expect steric and electronic clashes. **(C)** Representative, raw DSF curves for Fig 2C. See the text and Figure 2 for details. **(D)** Clustal alignment of the C-terminal regions of human Hsp70 (HSPA1A) and tauC3, highlighting the Ser/Thr residues and especially the P6 position (black box).

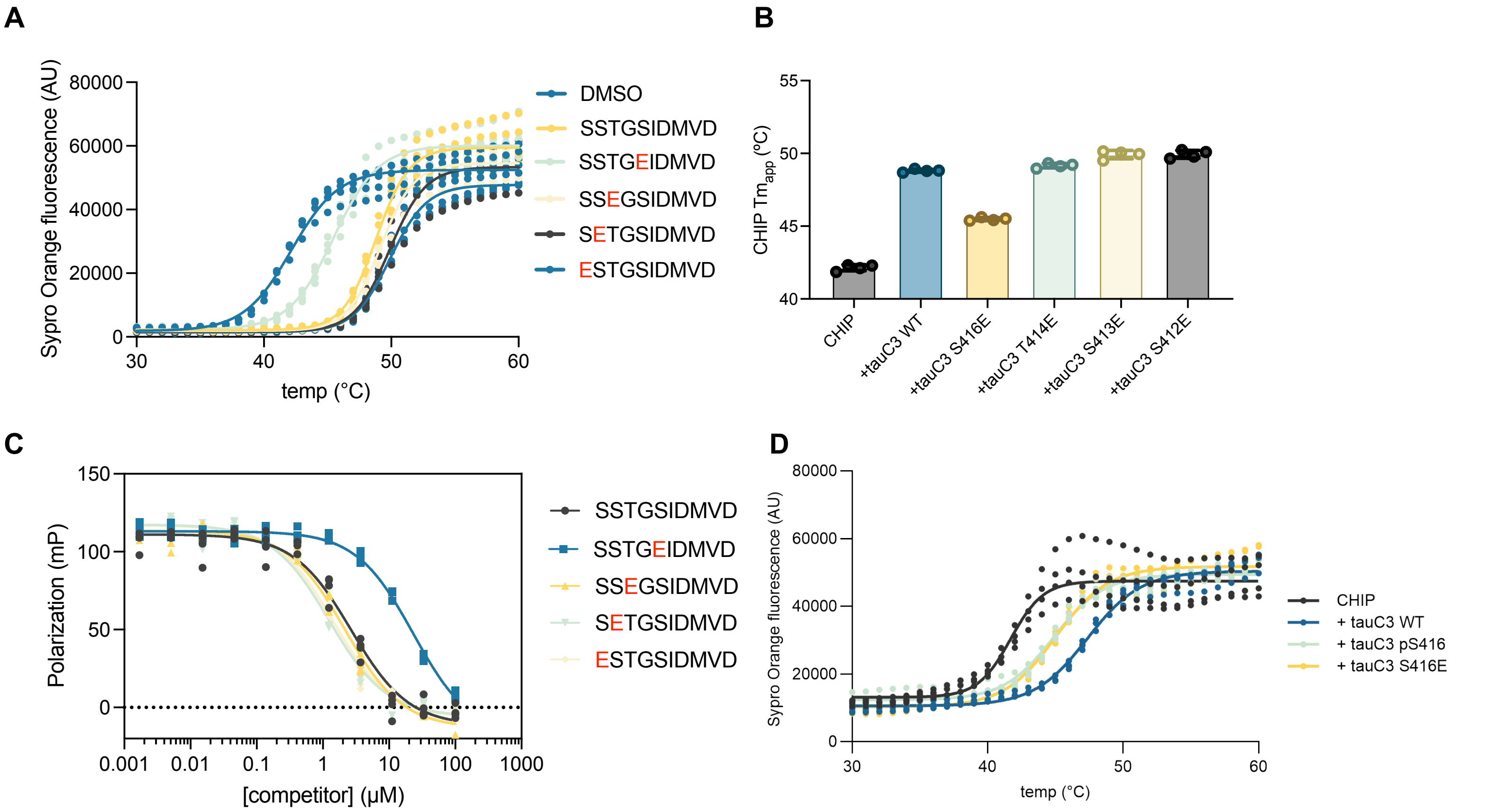

Supplemental Fig. S3. Pseudo-phosphorylation at serine 416 is sufficient to inhibit CHIP binding. (A) DSF melt curves for CHIP in the presence of various 10-mer tauC3 phosphomimetic peptides. Assay was performed in technical quadruplicate and melt curves were fit with a Boltzmann sigmoid. **(B)** Apparent melting temperatures (Tm_{app}) of CHIP WT in the absence or presence of 10-mer tau peptides as derived from (A). **(C)** Competition FP experiment showing displacement of fluorescent tracer from the CHIP TPR domain by various 10-mer tau peptides. Samples were performed in technical quadruplicate. **(D)** Representative, raw DSF curve for Fig 2D. See Figure 2 and the text for details.

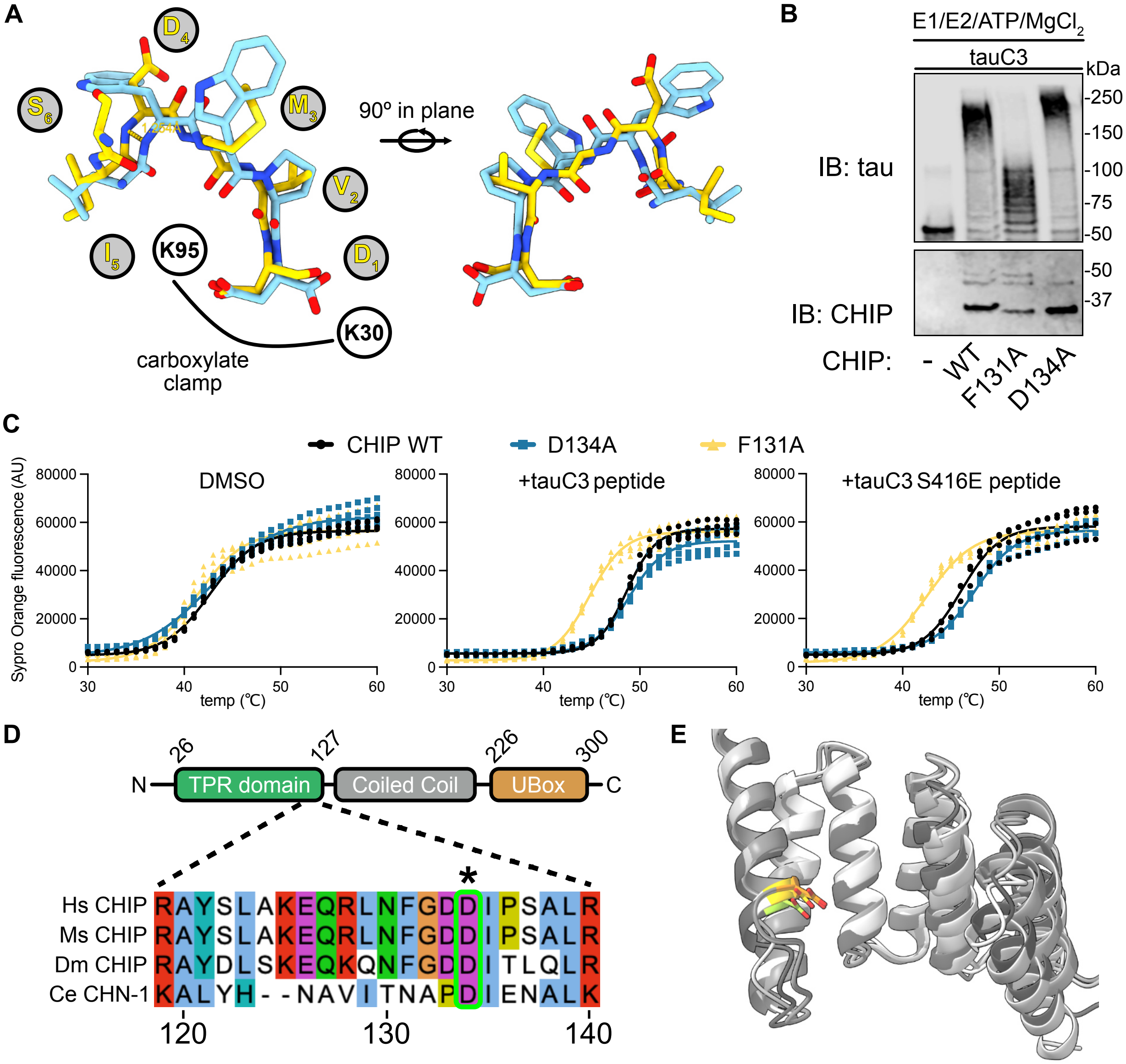

Supplemental Fig. S4. Mutation of a conserved residue in CHIP (CHIP D134A) partially restores activity on tauC3 pS416. **(A)** Structural alignment of tauC3 peptide (yellow) with an optimized CHIP peptide (blue) bound to the CHIP TPR (PDB: 6NSV). Residues 10-7 from the tauC3 peptide are omitted for clarity. TauC3 residues 1-6 are noted in gray circles. Location of the CHIP carboxylate clamp residues are denoted in white open circles. The 1.254 Å shift in the backbone register is shown on the overlay. **(B)** In vitro ubiquitination of tauC3 by various CHIP mutants. A single time point was collected, quenched in SDS-PAGE loading buffer, and analyzed by western blot. **(C)** DSF melt curves for various CHIP mutants in the absence or presence of various 10-mer tauC3 peptides. Assay was performed in technical quadruplicate and melt curves were fit with a Boltzmann sigmoid. **(D)** Cartoon depicting the domain architecture of the CHIP monomer, with region of the TPR domain bearing D134 highlighted below. Conservation of D134 across evolution is highlighted in green with asterisk. (Hm = H. sapiens; Ms =M. musculus; Dm =D. melanogaster; Ce = C. elegans). **(E)** Structural overlay of CHIP TPR domain across evolution. AlphaFoldv2.0 structures from human, mouse, fruit fly, and nematode CHIP are shown in various shades of gray, with D134 highlighted in various shades of yellow.

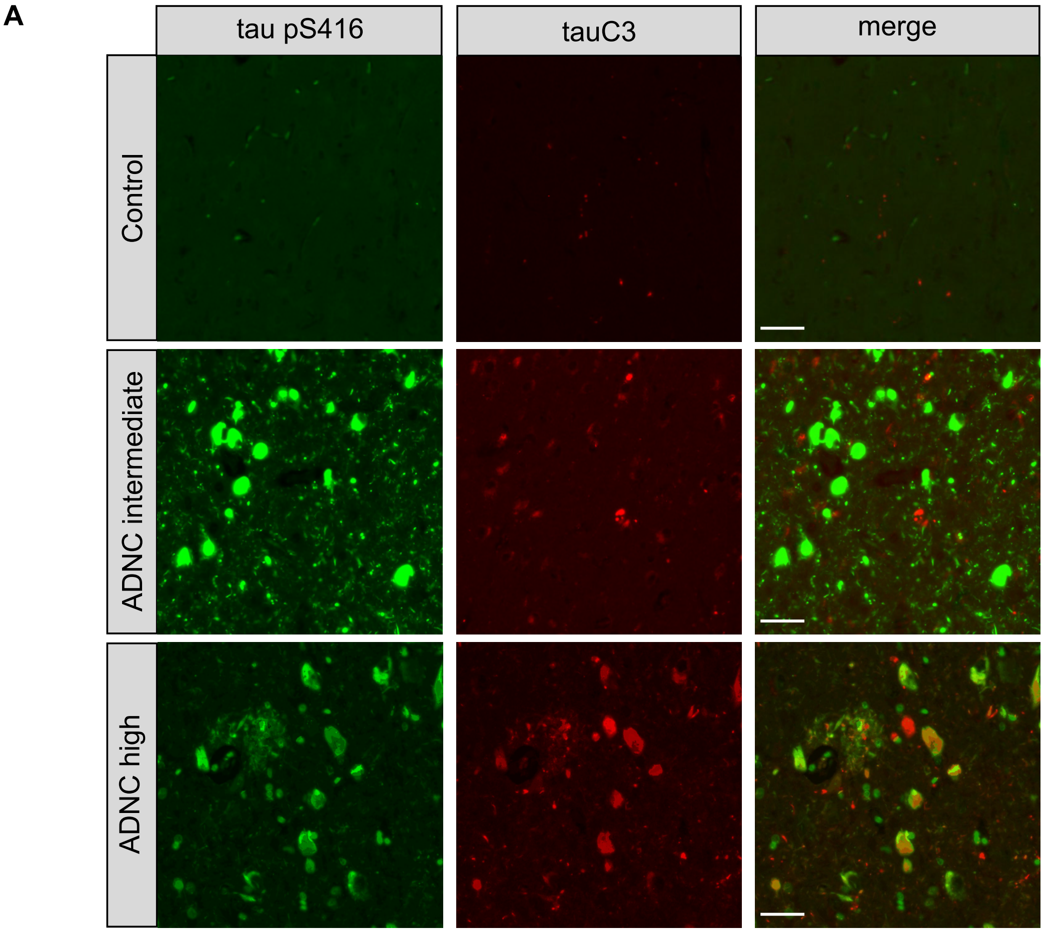

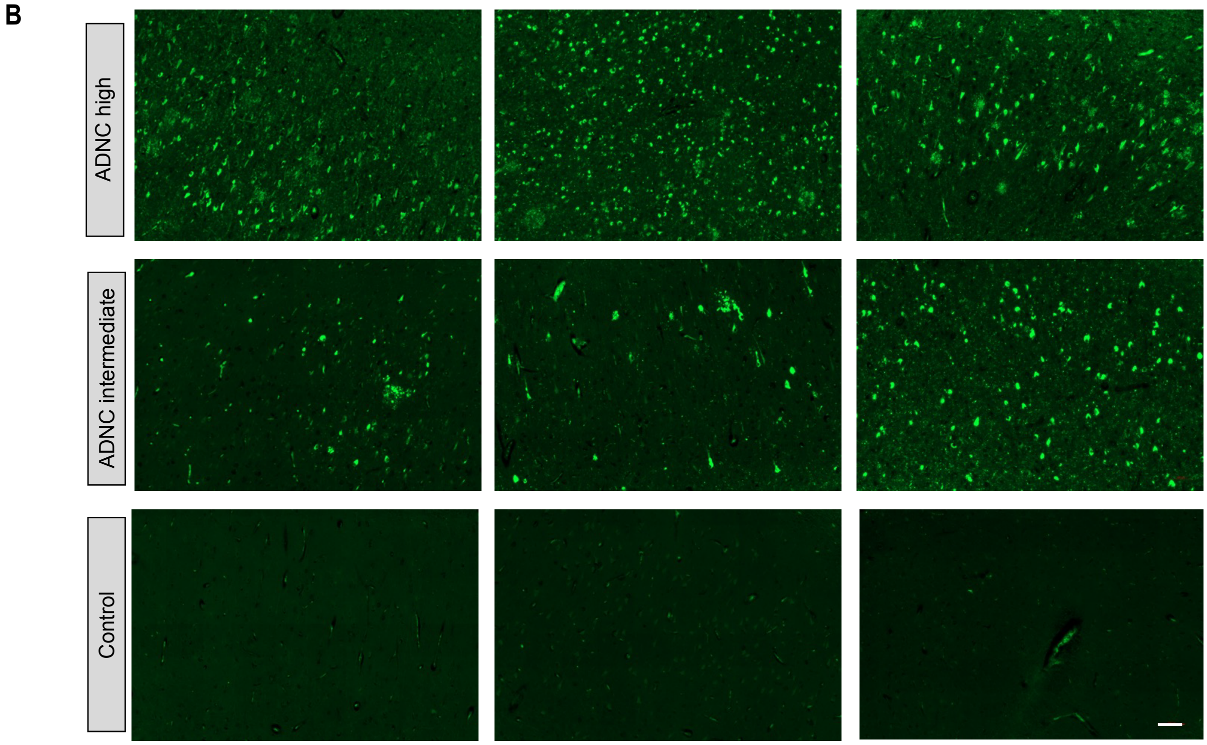

Supplemental Fig. S5. Additional examples of pS416 accumulation in AD and co-localization with tauC3. (A) Representative immunofluorescence micrographs from the subiculum of human patient samples across increasing ADNC score. Tau pS416 is shown in green, while tauC3 staining is shown in red. Scale bar = 50 μM. (B) Representative immunofluorescence micrographs from the subiculum of human patient samples, stained with AT8 antibody to confirm tau pathology.

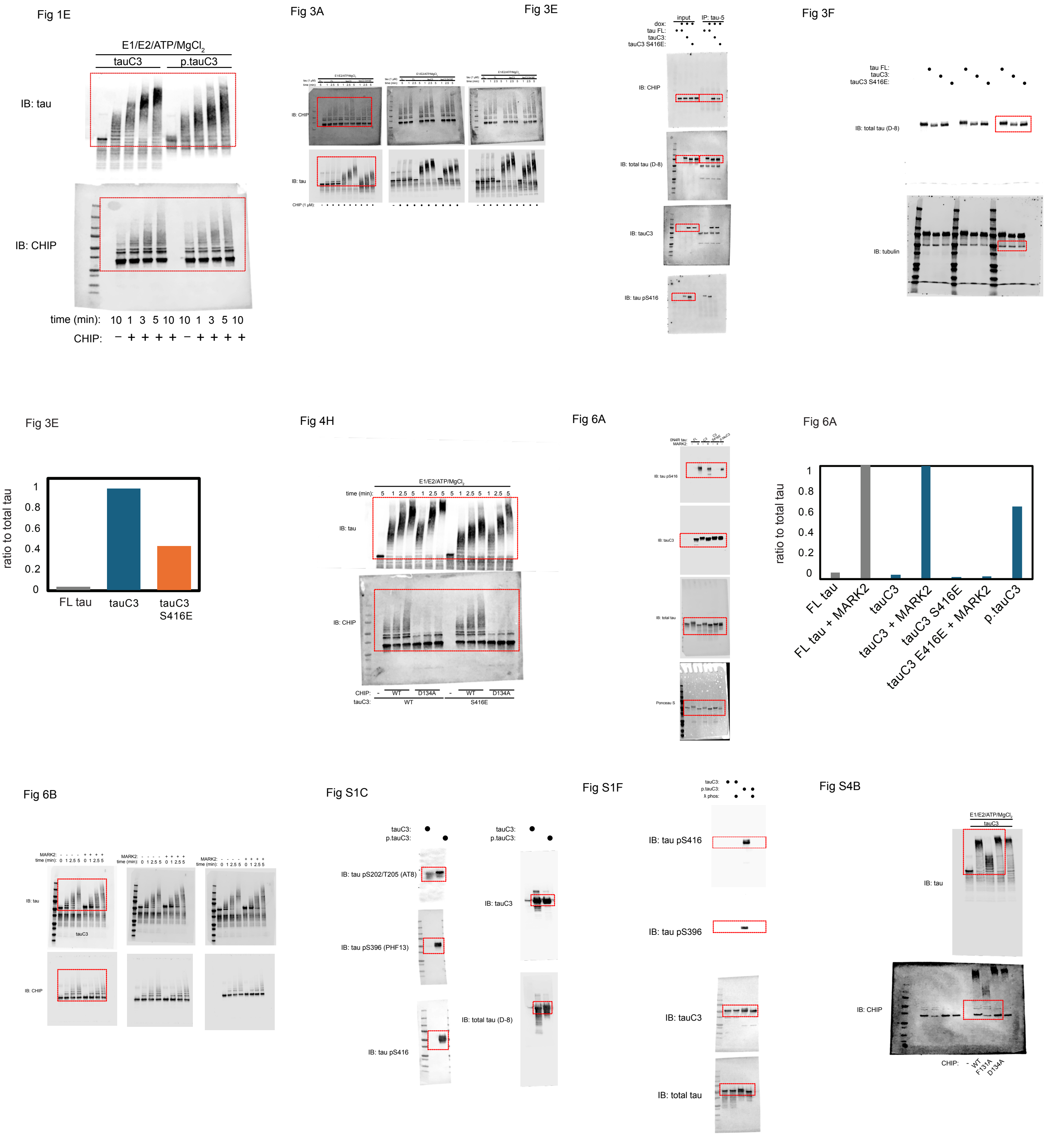

Supplemental Figure S6. Raw, uncropped blots and quantifications of select Western blots. Collection of raw, uncropped blots and image quanitifications, with the corresponding panel indicated above each image.
