# Supplementary material for: Phosphorylation of tau at a single residue inhibits binding to the E3 ubiquitin ligase, CHIP
Source: Nat Commun. 2024 Sep 12;15:7972. doi: 10.1038/s41467-024-52075-1 (PMC11393453; doi:10.1038/s41467-024-52075-1)
Supplement: Supplementary file 4 — Description of Additional Supplementary Files [file 41467_2024_52075_MOESM4_ESM.pdf]

Supplementary Data 1

Mass Spectrometry Results
